# Frailty Is a Better Predictor than Age of Mortality and Perioperative Complications after Surgery for Degenerative Cervical Myelopathy: An Analysis of 41,369 Patients from the NSQIP Database 2010–2018

**DOI:** 10.3390/jcm9113491

**Published:** 2020-10-29

**Authors:** Jamie R. F. Wilson, Jetan H. Badhiwala, Ali Moghaddamjou, Albert Yee, Jefferson R. Wilson, Michael G. Fehlings

**Affiliations:** 1 Nebraska Medical Center, University of Nebraska Medical Center, Omaha, NE 68198, USA; jamie.wilson@unmc.edu; 2 Spine Program, Department of Surgery, University of Toronto, Toronto, ON M5T 2S8, Canada; jetan.badhiwala@gmail.com (J.H.B.); Ali.Moghaddamjou@one-mail.on.ca (A.M.); Albert.Yee@sunnybrook.ca (A.Y.); WilsonJeff@smh.ca (J.R.W.); 3 Division of Neurosurgery, Krembil Neuroscience Centre, Toronto Western Hospital, University Health Network, Toronto, ON M5T 2S8, Canada

**Keywords:** degenerative cervical myelopathy, frailty, age, mortality, complications

## Abstract

Background: The ability of frailty compared to age alone to predict adverse events in the surgical management of Degenerative Cervical Myelopathy (DCM) has not been defined in the literature. Methods: 41,369 patients with a diagnosis of DCM undergoing surgery were collected from the National Surgical Quality Improvement Program (NSQIP) Database 2010–2018. Univariate analysis for each measure of frailty (modified frailty index 11- and 5-point; MFI-11, MFI-5), modified Charlson Co-morbidity index and ASA grade) were calculated for the following outcomes: mortality, major complication, unplanned reoperation, unplanned readmission, length of hospital stay, and discharge to a non-home destination. Multivariable modeling of age and frailty with a base model was performed to define the discriminative ability of each measure. Results: Age and frailty have a significant effect on all outcomes, but the MFI-5 has the largest effect size. Increasing frailty correlated significantly with the risk of perioperative adverse events, longer hospital stay, and risk of a non-home discharge destination. Multivariable modeling incorporating MFI-5 with age and the base model had a robust predictive value (0.85). MFI-5 had a high categorical assessment correlation with a MFI-11 of 0.988 (*p* < 0.001). Conclusions and Relevance: Measures of frailty have a greater effect size and a higher discriminative value to predict adverse events than age alone. MFI-5 categorical assessment is essentially equivalent to the MFI-11 score for DCM patients. A multivariable model using MFI-5 provides an accurate predictive tool that has important clinical applications.

## 1. Introduction

Degenerative cervical myelopathy (DCM) is characterized by progressive compression of the spinal cord in the cervical canal, producing debilitating neurological deficits in upper limb function, gait instability, sphincteric disturbance, and ultimately spastic quadriparesis. It is the most common cause of adult spinal cord dysfunction worldwide and its prevalence increases significantly with age [1]. With the projected shift in demographics over the next 30 years, the burden of (potentially treatable) neurological dysfunction in the elderly has become a major public health concern of the 21st century [1,2,3,4]. The Institute of Medicine has declared that DCM includes 3 of the top 100 national priorities for comparative effectiveness in research, and efforts to address the rise of disability from DCM have been implemented worldwide, including the establishment of international consensus treatment guidelines [5,6,7].

DCM is an umbrella term that encompasses a number of degenerative pathologies that include osteoarthritis (spondylosis), ligament disease (ossification of the posterior longitudinal ligament (OPLL), ligament hypertrophy), and degenerative listhesis or instability. These entities have pronounced effects on the functional abilities and quality of life of impaired individuals, which may be comparable to serious health conditions, such as cancer or heart disease [8]. The mainstay of treatment for DCM is decompressive surgery, which arrests the progression of the disease and provides a sustained and meaningful improvement in functional and quality of life measures [2,3,7,9,10,11,12,13]. Clinical factors such as duration of symptoms prior to surgery and severity of baseline functional impairment correlate strongly with the chances of a substantial clinical benefit after intervention [9,11,12,14,15].

Decisions regarding the best application of surgical intervention for DCM have become an important focus of clinical study [3,6]. In the elderly population, this issue becomes complex as the potential impact of preventing neurological disability in the elderly needs to be balanced against the healthcare costs and complication profile [4,6,7,13]. Although increasing age is associated with poorer surgical outcomes, in DCM patients many studies have shown sustained long-term functional and quality of life improvements after surgery [4,10,11,15,16,17]. Moreover, when elderly patients are matched for co-morbidities and baseline functional impairment, their complication profile is equivalent [3]. This has led to the evolving opinion that age alone has become less relevant for the purpose of estimating perioperative risk profile and prognosis after surgery [18,19,20,21,22].

Efforts to move past age alone as a predictor of outcomes has led to the development of measures of physiological reserve (or ‘frailty indices’). The most commonly cited index in spine surgery is the Modified Frailty Index (11-point or 5-point, Table 1) [23,24,25,26] which is derived from the original 70-point Canadian Study of Health and Aging Frailty Index (CSHA-FI) [27]. Other measures include the American Society of Anesthesiologists Grading scale (ASA) and the modified Charlson Co-morbidity Index (mCCI). ASA is a well-established subjective estimate of overall illness severity that has an uncertain role in the prediction of perioperative outcomes after spine surgery [18]. The mCCI, like the MFI, is also matched to NSQIP variables but produces different weightings according to increasing age and certain co-morbidities. Although used widely in general surgery, the mCCI has received limited application in spine surgery [19,20,28]. In 2017, Shin et al. published a study of 6965 patients who underwent either anterior cervical discectomy or posterior cervical fusion, and showed frailty (as measured by the 11-point MFI) was associated with an increased risk of adverse events [23]. However, this study did not distinguish between radiculopathy or myelopathy patients, and it has been previously demonstrated that myelopathy patients have a higher risk of perioperative complications compared to patients with purely radicular symptoms [24]. To date, no study exists that models the impact of frailty specifically on surgical DCM patients, or that compares the discriminatory ability of different measures of frailty.

The objectives of the current study were to (1) define the effect of age on the perioperative outcomes of mortality, unplanned readmission/reoperation, major complication, length of stay and discharge to non-home destination for patients undergoing surgery for DCM, (2) directly compare measures of frailty in the same cohort to determine which factor exhibits a greater influence on the observed outcomes, and (3) define the potential correlation between MFI-5 and MFI-11 in DCM patients. We hypothesize that after adjustment for common surgical factors, frailty is a better predictor of perioperative complications compared to age alone. Frailty as a predictor of perioperative complications would have important implications for the clinical management of elderly patients with DCM.

## 2. Experimental Section

### 2.1. Data Source

The data source for this study was the American College of Surgeons (ACS) National Surgical Quality Improvement Program (NSQIP) database, for years 2010 through 2018 inclusive. The NSQIP datasets encode surgical procedures by Current Procedural Terminology (CPT) codes and diagnoses by International Classification of Diseases, Ninth/Tenth Revision, Clinical Modification (ICD-9/10-CM) codes. NSQIP collects pre-operative through 30-day post-operative data on randomly assigned patients at participating hospitals. Quality and reliability of the data are ensured through rigorous training of data abstractors and inter-rater reliability audits of participating sites [25].

### 2.2. Patient Population

Eligible patients who had a primary diagnosis of DCM (ICD-9-CM 721.1 or 722.71; ICD-10-CM M47.12 or M50.00, M50.01, M50.02, M50.03) and underwent a cervical decompression and fusion operation, including anterior (CPT 22551, 22554, 63081) and/or posterior (CPT 22600, 63051, 63020) approach. ICD-9/10-CM and CPT codes used for this study are summarized in Table 2.

### 2.3. Baseline Characteristics

Data relating to baseline demographic characteristics and comorbidities were extracted. The number of operated levels was determined by searching the “other procedure” fields for CPT add-on codes specifying each additional level fused (CPT 22552, 22614). Surgical approaches were separated into anterior, posterior, or combined as a categorical variable.

### 2.4. Calculation of Frailty Indices

The MFI-11 was calculated according to established mapping of existing variables included in the NSQIP database (see Table 1). A total score between zero and one was calculated by dividing the number of variables present (for functional status, partial or complete dependency = 1) by 11. Afterwards, 0.09 was categorized as “Pre-Frail”, 0.18 as “Frail”, and 0.27 and above as “Severely Frail”, in line with previously established standards [26,29]. A modified frailty index (mFI-5) was derived according to the standard methodology described by Searle et al. [30], calculated using NSQIP variables [31]. Specifically, five factors within the NSQIP (functional dependence, diabetes, history of chronic obstructive pulmonary disease (COPD), history of congestive heart failure, and hypertension) map to the original Canadian Study of Health and Aging (CSHA) Frailty Index [27]. For every patient, each of these five factors (deficits) were coded as absent (0) or present (1). The mean score across all deficits was calculated, resulting in an index ranging from 0 (least frail) to 5 (most frail), with a score of 1 as “Pre-Frail”, 2 as “Frail”, and 3 or more as “Severely Frail” as categorical variables. ASA score was taken directly from the NSQIP database for each patient. The mCCI score was calculated according to previous methods mapped directly from the corresponding NSQIP variables [18], creating a score from 0 to 23 depending on the age and presence of defined co-morbidities.

### 2.5. Outcomes

Outcomes evaluated were 30-day mortality, unplanned readmission, unplanned reoperation, and major complication, as well as total hospital length of stay (LOS) and routine discharge (home). Major complication was a composite outcome of pneumonia, deep vein thrombosis (DVT), pulmonary embolism, myocardial infarction, cardiac arrest, wound infection or dehiscence, stroke, and sepsis.

### 2.6. Statistical Analysis

All statistical analyses were performed using Stata 16 (Stata Corp, College Station, TX, USA) with an a priori specified significance level of *p* = 0.05 (two-tailed). Descriptive statistics were by mean and standard deviation (SD) for continuous variables as well as count and percentage for categorical variables.

The effect of age, MFI-5, MFI-11, CCI, and ASA were each analyzed by univariate analysis using simple logistic regression for dichotomous outcomes or linear regression for continuous outcomes. Effect sizes were summarized by odds ratio (OR) (dichotomous outcomes) or beta coefficients (continuous outcomes) and associated 95% confidence intervals (95% CI). The independent effect of age and frailty on outcomes was further evaluated by multivariable regression. Again, for each outcome, a logistic or linear regression model was constructed that included both variables and additionally adjusted for sex, type of fusion, and number of levels as covariates. To weigh the relative importance of age versus frailty in predicting each outcome, standardized regression coefficients were calculated and their magnitudes directly compared. The margins of interaction of the final model of age (by decade) and frailty (continuous variable) were calculated for all adverse events to assess how the burden of frailty was affected by increasing age.

To compare the discriminative ability of age and the various indices of frailty, receiver operating characteristics (ROC) curve analysis was performed for each dichotomous outcome reported. This was done first using a base model that included the type of approach, number of operated levels and gender, and then with the addition of age and each index of Frailty, before comparing to the final multivariable model incorporating age, MFI-5, and the base model. The Kappa correlation coefficient was calculated to assess the discriminative ability of the MFI-5 to predict the categorical assessment of frailty compared to the MFI-11 assessment.

### 2.7. Ethics Approval

Institutional Review Board approval was not required for this study, which relied on de-identified data derived from a national administrative healthcare dataset.

## 3. Results

A total of 41,369 patients with the ICD-9/ICD-10 diagnostic code for DCM were identified from the NSQIP database. The mean age was 56.6 (56.5–56.7) years with a range of 18–90, and 46% of patients were female. The majority of patients were Caucasian, although ethnicity metrics were not captured in 9.84% of patients. Data on the surgical approach, based on validated CPT codes, was available for 34,287 patients. Anterior, posterior and combined anterior-posterior surgical approaches were all included; however, 79.86% of the patients underwent anterior surgery. Furthermore, the study included single and multi-level disease, but the majority of cases were single or two-level pathology (39.24% and 31.64% respectively). The ASA grade and mCCI scores were calculated in all patients, and the median was 3 and 2, respectively. MFI-11 was calculated for 11,758 patients with 39.55% and 37.47% in the “Not Frail” or “Pre-Frail” categories. MFI-5 scores were calculated for 41,140 patients, with 44.68% “Not Frail” and 36.03% “Pre-Frail”. Complete descriptive statistics for all indices are listed in Table 3.

### 3.1. Univariate Analysis of Age and Frailty Indices on Outcomes

Univariate analysis demonstrated that age, frailty (MFI-5 or MFI-11), ASA, and mCCI were significantly predictive of perioperative mortality, major complication, unplanned readmission, unplanned reoperation, length of hospital stay, and discharge to non-home destination (Table 4). The OR effect size of age increases by decade for mortality, major complication, length of stay, and discharge to non-home destination (see Table 5). Based on categorical analysis of frailty tiers, increasing frailty was significantly associated with increased risk of all adverse events, increased length of stay and non-home discharge. The effect size of the MFI-5 index on all outcomes was greater than the MFI-11. Categorical correlation between frailty tiers calculated by the MFI-11 and MFI-5 was strongly significant with a kappa coefficient of 0.96 (97.58% agreement) and a spearman rank correlation of 0.988 (*p* < 0.001).

### 3.2. Multivariable Analysis Adjusting for Approach, Number of Levels Operated and Gender

The results from the multivariable regression analysis (adjusting for sex, surgical approach, and number of operated levels) demonstrate that age and/or frailty (as measured by the mFI) both have significant effects on the outcomes of patient mortality, unplanned readmission, unplanned reoperation, major complication, length of stay, and discharge home (see Table 6). Both increasing age and higher frailty index score were significantly associated with an increased risk of mortality (*p* < 0.001), but the effect size (as demonstrated by the beta coefficient) was greater for frailty (0.53) as compared to age (0.30). The effect size of frailty on the risk of a major complication event and length of stay was also greater than age, but increased frailty and increased age demonstrated equivalent effect sizes on the chance of a routine home discharge (−0.30 vs. −0.28). Increased age appeared to have a greater influence on the risk of unplanned readmission (0.17 compared to 0.14), however, age was not found to have any significant influence on the risk of unplanned reoperation (0.03; *p* = 0.676) and the effect size for frailty was of a magnitude 5.6 times greater (0.17; *p* = 0.016). Similar to the univariate analysis, increasing frailty was associated with a significantly increased risk of all outcomes, with ‘Severely Frail’ patients demonstrating an effect size 4–5 times larger for some outcomes (mortality, major complication, unplanned reoperation).

ROC area under the curve (AUC) analysis demonstrated the discriminative ability of the base model could be improved across all outcomes with the addition of age or an index of frailty (See Table 7). MFI-5 and CCI appeared to provide the best discriminative ability when added to the base model compared to the other measures of frailty. However, the final multivariable model demonstrated superior discriminative ability for all outcomes above all of the individual frailty measures + base models tested with an AUC range from 0.76–0.84.

## 4. Discussion

As the burden of age-related degenerative spine conditions becomes an ever-greater public health priority, DCM has the potential to be a major cause of preventable neurological disability and poor quality of life across the world [4,8,16]. Decompressive surgery remains the only treatment proven to arrest or reverse the dysfunction caused by myelopathy and has a proven benefit in the elderly population [3,7,10,32]. The diagnosis of DCM and identification of suitable surgical candidates is therefore of paramount importance, and for this reason, identifying tools to aid risk stratification and predict potential outcomes after surgery is a major focus of clinical research [6,9,13,14].

The evidence for decompressive surgery to improve functional and quality of life impairment in DCM is strong [7,10,32]. Reports on the effect of age on the outcomes after DCM surgery have been variable, with many supporting the notion that increasing age is associated with negative clinical outcomes, whether increased complication rates or functional outcomes [12,13,15,33]. A recent ambispective study has demonstrated a clear and sustained benefit for surgery in DCM for both functional and quality of life outcomes in patients over the age of 70, albeit with an order of magnitude less than their younger surgery- and co-morbidity-matched cohort [3]. The suggested mechanism for this discrepancy has traditionally been the burden of age-related co-morbidities influencing clinical outcomes and reduction of physiological reserve, which has been manifested as measures of frailty or frailty indices in recent years. This hypothesis has been further substantiated with the correlation of worsening frailty to increased complication rate after spine surgery, and poorer recovery after spinal cord injury [28,34,35]. However, despite DCM being the most common indication for cervical spine surgery in North America, the effect of frailty on the outcomes after DCM surgery has not been investigated [16].

This study is one of the most comprehensive investigations of perioperative adverse events after surgery for DCM, and is the first to present a direct comparison of age and frailty indices. Concepts of frailty, although distinct from risk stratification, are increasingly incorporated in the pre-operative assessment as a superior and comprehensive alternative to age alone. Although it is no surprise that increasing age leads to an increased risk of adverse events, it appears the burden of frailty can have an effect up to 28 times the magnitude compared to age alone. On univariate analysis, increasing frailty (MFI-5/MFI-11) had the largest effect size for mortality, major complication and discharge to non-home destination. This effect remained in the multivariable model, but the effect size was largest for mortality and unplanned reoperation. These findings suggest that physiological reserve is much more of a driver of perioperative complications when compared to age alone. This is consistent with previous studies on spine surgical patients, but also other surgical domains [36,37]. The degree to which frailty appears to have an influence on unplanned reoperation rates has a sound pathophysiological basis. Given the usual indications for early reoperation are compressive hematoma, infection, and hardware failure, patients who have a higher mFI score are more likely to develop post-operative infections and a higher risk of osteopenia/osteoporosis.

**Figure 1:**
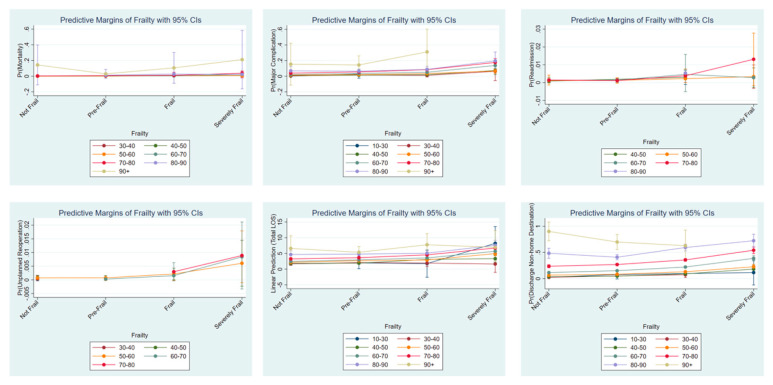
Predictive margins of the final multivariable model for each frailty level stratified per decade of age, for each outcome studied. Decades of age were removed if co-linearity was present. 95% CI, 95% Confidence Interval.

Frailty significantly affected the risk of perioperative adverse events for patients of all ages, but the burden of frailty becomes greater with increased age. This effect is not linear, as demonstrated by the margins of interaction between age (by decade) and categorical level of frailty (see Figure 1). This effect (for readmission, reoperation, length of stay, and discharge to non-home destination) begins after the age of 60, which is in line with previously reported studies [32,34,35].

The mCCI proved a significant predictor of all adverse events on univariate and multivariable analysis, and the effect size remained similar for both. This may suggest that it could be a useful tool in the assessment of DCM patients. However, the role of the mCCI in multivariable modeling is unclear due to the inclusion of age-related modifiers. The mCCI incorporates increasing age as a contributor to the overall score, and therefore does not provide an age-independent measure of frailty. It cannot therefore be a definitive frailty assessment when incorporated into a multivariable model that includes age as a continuous variable. The ASA score also proved to be significantly predictive of adverse events on both univariable and multivariable analysis. However, the ASA score is notoriously subjective and its practical use for pre-operative adverse event prediction modeling has not been substantiated.

There was strong correlation between the MFI-11 and MFI-5 assessment of frailty tiers, which has been echoed in previous articles in other spine pathologies [28]. The effect size of the odds ratio or regression coefficients were larger when the MFI-5 was used compared to the MFI-11 for all dichotomous outcomes and length of hospital stay. This would suggest that to achieve an assessment of “Frail” or “Severely Frail” with the MFI-5 this would indicate a greater degree of frailty compared to the MFI-11 equivalent. This is strong evidence that the use of the MFI-5 is an effective determinant of frailty and further substantiates the MFI-5 as the standard of choice for frailty assessment for DCM patients. This has important implications for the use of MFI-5 in clinical practice and for future studies into the effect of frailty in adult spine surgery.

There are limitations to the current study. The NSQIP database carries metrics regarding peri-operative events, but no long term follow up or outcome measures are included. This restricts the analysis to short term follow up only, and therefore long-term outcomes cannot be extrapolated. However, the validity of short-term complication rates and their use for pre-operative decision-making in spine surgery is well published. In a similar vein, there is no measure of baseline functional impairment and therefore there is no way to eliminate the effect of myelopathy severity on the surgical approach, or other covariates applied to the regression model. Pre-operative neurological function is a key predictor of the functional outcomes of DCM surgery, however its relationship to perioperative adverse events and mortality is less clear. It is known that myelopathy patients have increased perioperative complications compared to radiculopathy [26], but there is a paucity of evidence of the effect of functional impairment on perioperative outcomes in DCM.

There was an observed difference in the number of patients that were able to have the MFI-11 calculated (*n* = 11,758) compared to the MFI-5 (*n* = 41,140). This difference arose arbitrarily due to a reduction in the demographic information collected by NSQIP from 2015 onwards. This difference in numbers has the potential to skew the effects and effect size of the results presented. The authors argue that given the categorical correlation of frailty was excellent between MFI-5 and MFI-11 (97.58% agreement), the large size of the cohorts, the strength of the a priori statistical frameworks and the levels of significance observed, the risk of statistical inaccuracy is low.

The study is a retrospective analysis of prospectively collected nationwide hospital inpatient registry data. As such, it lacks the protection from confounders and bias from a true prospectively-collected data set. However, uniformity across the data collection and homogeneity amongst the selected cohort does reduce the impact of any latent confounding. The cohort selection relied on ICD-9 & 10 diagnostic codes, and therefore the potential risk of not capturing patients if they were inappropriately coded is present. Patients were removed from the final analysis if no reliable CPT code data could identify the surgical approach or number of levels, which led to a reduction in the overall number of patients in the multivariable model. It is unlikely that these occurrences have affected the findings of the study given the levels of significance seen on univariable and multivariable analysis.

The authors note that DCM carries a significant heterogeneity in presenting symptoms ranging from mild sensory disturbance in the fingers through to gait instability and eventual quadriparesis. Traditional measures of frailty, in particular those based on 5- or 11-point scales as used in this study, may not be as accurate when compared to other pathologies not affecting gait or upper limb function. Therefore, developing more appropriate indices of frailty specific to DCM should be a future research priority. Also, the authors wish to state that the purpose of this study is not to prove patients who have an increased frailty score should not be offered surgical management. In contrast, this study should be used to provide further clarification of the factors that go into informed decision-making, which should be a shared process between the patient and clinician. The concept that frailty could potentially be viewed as a modifiable risk factor is also emerging, however evidence that improving frailty index scores prior to planned spine surgery to reduce perioperative complications is not conclusive at this stage [38].

## 5. Conclusions

Increasing frailty appears to have a greater influence on the risk of perioperative mortality, risk of major complication, unplanned readmission, unplanned reoperation, and longer duration of hospital stay when directly compared to age alone for patients undergoing surgery for DCM. The MFI-5 is a robust predictor of adverse outcomes after DCM surgery and is equivalent to the traditional MFI-11 when categorizing patients as ‘Pre-Frail’, ‘Frail’, or ‘Severely Frail.’ Multivariable models incorporating MFI-5 with age, approach, number of levels, and gender provide an accurate and reliable method of predicting outcomes from DCM surgery. Future work should focus on how to delineate the association of frailty with long term functional and quality of life outcomes after DCM surgery.

## Figures and Tables

**Table 1:** NSQIP clinical variables matched from the CSHA-FI used to construct the 11- and 5- item modified frailty index (MFI-11, MFI-5), compared with the modified Charlson Co-morbidity Index (mCCI).

NSQIP Variables		CSHA-FI	mCCI (Weighting)
MFI-11	MFI-5		
Functional health status prior to admission	Functional health status prior to admission	Changes in daily activities	Ascites/Esophageal Varices (3)
Diabetes Mellitus	Diabetes Mellitus	Diabetes Mellitus	Diabetes Mellitus (1)
History of Severe COPD	History of Severe COPD	Respiratory problems	History of Severe COPD (1)
Hypertension requiring medication	Hypertension requiring medication	Arterial hypertension	Renal Failure (2)
Congestive Heart failure within 30 days of admission	Congestive Heart failure within 30 days of admission	Congestive heart failure	Congestive heart failure (1)
Myocardial Infarction within past 6 months prior to surgery		Myocardial Infarction	Prior Myocardial Infarction (1)
Previous Cardiac Surgery OR Angina <1 month prior to surgery		Cardiac problems	Disseminated Cancer (6)
Impaired sensorium		Clouding or delirium	
History of TIA or Cerebrovascular Accident with no deficits		Cerebrovascular problems	Prior TIA or Cerebrovascular Accident (1)
Cerebrovascular Accident with deficits		History of stroke	Hemiplegia (2)
Previous intervention for peripheral vascular disease OR Rest pain/Gangrene secondary to peripheral vascular disease		Decreased peripheral pulses	Peripheral Vascular Disease (1)
			40 years old or less (0)
			41–50 years old (1)
			51–60 years old (2)
			61–70 years old (3)
			71 years old+ (4)

NSQIP, National Surgical Quality Improvement Program; CSHA-FI, Canadian Study of Health and Aging Frailty Index; mCCI, modified Charlson Co-morbidity Index; COPD, chronic obstructive pulmonary disease; MFI, modified frailty index; OR, Odds Ratio; TIA, transient ischemic attack.

**Table 2:** List of ICD-9, ICD-10, and Current Procedural Terminology (CPT) codes used to determine diagnosis, operative approach and number of operated levels.

Coding System	Code	Description
ICD-9-CM	721.1	Cervical spondylosis with myelopathy
	722.71	Intervertebral disc disorder with myelopathy, cervical region
ICD-10-CM	M47.12	Other spondylosis with myelopathy, cervical region
	M50.00, M50.01, M50.02, M50.03	Cervical disc disorder with myelopathy
CPT	22551	Arthrodesis, anterior interbody, including disc space preparation, discectomy, osteophytectomy and decompression of spinal cord and/or nerve roots; cervical below C2+ Each additional interspace 22552
	22600	Arthrodesis, posterior or posterolateral technique, single level; cervical below C2 segment+ Each additional vertebral segment 22614
	22856	Cervical arthroplasty (anterior)
	63081	Cervical corpectomy (anterior)
	63001	Posterior cervical laminoplasty
	63045, 63015	Posterior cervical laminectomy

ICD, International Classification of Diseases; CM, Clinical Modification; CPT, Current Procedural Terminology.

**Table 3:** Patient demographics and descriptive statistics.

**Age Mean (95% CI)**	**56.6 (56.5–56.7)**
Distribution (*n*):	
18–30	627
30–40	3728
40–50	8858
50–60	12,427
60–70	9966
70–80	4745
80–90	931
90+	87
**Gender**	
Male	22,191 (54%)
Female	19,167 (46%)
**Ethnicity**	
White	30,778 (74%)
Black/African American	5260 (13%)
Asian	897 (2.1%)
American Indian	222 (0.5%)
Native Hawaiian/Pacific Islands	141 (0.4%)
Unknown	4071 (10%)
**Approach (where defined)**	
Anterior	27,380 (80%)
Posterior	5945 (17%)
Combined	962 (3%)
**Distribution of Frailty**	
MFI5	
Not frail	18,482 (45%)
Pre-frail	14,904 (36%)
Frail	6816 (16%)
Severely Frail	1167 (3%)
MFI11	
Not frail	4650 (40%)
Pre-frail	4406 (37%)
Frail	2239 (19%)
Severely Frail	463 (4%)
mCCI score	
0	4125 (10%)
1–2	17,613 (43%)
3–4	14,670 (35%)
5–6	4645 (11%)
>6	316 (1%)
ASA	
1	1359 (3%)
2	19,289 (47%)
3	19,354 (47%)
4	1325 (3%)

MFI-5, MFI-11, Modified Frailty Index 5-point or 11-point; mCCI, Modified Charlson Co-morbidity Index; ASA, American Society of Anesthesiology Grade; 95% CI, 95% Confidence Interval.

**Table 4:** Univariate analysis for age, MFI-5, MFI-11, mCCI and ASA grade on the outcome of Mortality, Major Complication, Unplanned Readmission, Unplanned Reoperation, Length of Hospital Stay, and Discharge to non-home destination.

	Mortality	Major Complication (Pneumonia, DVT/PE, MI, Cardiac Arrest, Wound Infection/Dehiscence, Sepsis, CVA)	Unplanned Readmission	Reoperation	Length of Hospital Stay (Regression Coefficient)	Discharge to Non-Home Destination
Age	1.09 (1.08–1.11) *	1.06 (1.05–1.06) *	1.05 (1.03–1.07) *	1.07 (0.99–1.04)		1.072 (1.070–1.075) *
MFI5						
Pre-frail	4.89 (2.72–8.79) *	2.40 (2.06–2.80) *	1.30 (0.71–2.40)	0.57 (0.22–1.51)	0.82 (0.70–.94) *	2.22 (2.07–2.38) *
Frail	8.37 (4.58–15.32) *	3.80 (3.22–4.48) *	3.40 (1.89–6.12) *	2.71 (1.26–5.86) *	1.67 (1.52–1.82) *	3.85 (3.56–4.12) *
Severely Frail	27.70 (14.29–53.69) *	11.63 (9.44–14.33) *	6.37 (2.80–14.50) *	8.57 (3.41–21.52) *	3.74 (3.42–4.06) *	8.67 (7.62–9.86) *
MFI11						
Pre-frail	4.45 (1.68–11.81) *	1.87 (1.45–2.41) *	1.11 (0.59–2.09)	0.49 (0.18–1.28)	0.80 (0.47–1.13) *	1.71 (1.53–1.91) *
Frail	7.11 (2.62–19.29) *	2.84 (2.17–3.72) *	2.75 (1.51–5.01) *	1.92 (0.88–4.22)	1.83 (1.42–2.24) *	2.89 (2.56–3.26) *
Severely Frail	20.51 (6.98–60.26) *	7.39 (5.30–10.31) *	3.74 (1.56–8.95) *	4.68 (1.77–12.38) *	4.39 (3.61–5.16) *	7.81 (6.38–9.56) *
mCCI	1.76 (1.62–1.90) *	1.51 (1.47–1.56) *	1.40 (1.25–1.58) *	1.32 (1.11–1.57) *	0.53 (0.50–0.56) *	1.59 (1.56–1.62) *
ASA	4.14 (3.12–5.50) *	3.60 (3.26–3.98) *	1.40 (1.25–1.85) *	1.32 (1.12–1.57) *	1.58 (1.50–1.67) *	3.35 (3.19–3.53) *

MFI-5, MFI-11, Modified Frailty Index 5-point or 11-point; mCCI, Modified Charlson Co-morbidity Index; ASA, American Society of Anesthesiology Grade; DVT, deep vein thrombosis; PE, Pulmonary Embolus; MI, Myocardial Infarction; CVA, Cerebrovascular Accident. * indicates the result is statistically significant (*p* value < 0.05).

**Table 5:** Univariate analysis by decade demonstrates increasing effect size (odds ratios) with increasing age for the outcomes of Mortality, Major Complication, Length of Hospital Stay, and Discharge Destination.

Age by Decade	Mortality	Major Complication (Pneumonia, DVT/PE, MI, Cardiac Arrest, Wound Infection/Dehiscence, Sepsis, CVA)	Unplanned Readmission	Reoperation	Length of Hospital Stay (Regression Coefficient)	Discharge to Non-Home Destination
30–40	0.0044 (0.0005–0.038) *	1.04 (0.41–2.70)	0.046 (0.0041–0.51) *	0.32 (0.067–1.50)	−0.13 (−0.59–0.32)	0.84 (0.56–1.25)
40–50	0.015 (0.047–0.046) *	1.55 (0.63–3.81)	0.097 (0.012–0.77) *	0.40 (0.14–1.16) *	0.11 (−0.33–0.55)	1.30 (0.90–1.90)
50–60	0.023 (0.0086–0.066) *	2.87 (1.17–6.97) *	0.12 (0.016–0.90) *	0.76 (0.33–1.78)	0.73 (0.29–1.15) *	2.23 (1.54–3.23) *
60–70	0.073 (0.028–0.19) *	4.93 (2.03–11.96) *	0.19 (0.025–1.43)	0.42 (0.15–1.15)	1.23 (0.79–1.67) *	4.29 (2.96–6.21) *
70–80	0.17 (0.065–0.43) *	9.03 (3.72–21.92) *	0.35 (0.046–2.61)	†	2.21 (1.75–2.66) *	9.36 (6.45–13.58) *
80–90	0.21 (0.074–0.63) *	11.69 (4.71–29.04) *	0.27 (0.029–2.70)	†	3.23 (2.68–3.78) *	20.73 (14.05–30.57) *
90+	†	19.90 (6.82–58.05) *	†	†	3.87 (2.65–5.09) *	52.24 (28.78–94.82) *

Correlation between frailty tiers calculated by the MFI-11 and MFI-5 was strongly significant with a kappa coefficient of 0.96 (97.58% agreement) and a spearman rank correlation of 0.988 (*p* < 0.001). † indicates that this decade was removed from analysis due to collinearity; * indicates the result is statistically significant (*p* value < 0.05).

**Table 6:** Multivariable analysis adjusting for age, gender, number of levels and surgical approach including one of either MFI-5, mFI-11, mCCI or ASA as the inter-changeable dependent variable.

	Mortality	Major Complication (Pneumonia, DVT/PE, MI, Cardiac Arrest, Wound Infection/Dehiscence, Sepsis, CVA)	Unplanned Readmission	Reoperation	Length of Hospital Stay (Regression Coefficient)	Discharge to Non-Home Destination
Age	1.08 (1.05–1.10) *	1.04 (1.03–1.05) *	1.02 (0.99–1.04)	0.98 (0.95–1.02)	0.021 (0.015–0.026) *	1.05 (1.05–1.06) *
MFI5						
score	2.03 (1.65–2.51) *	1.67 (1.54–1.80) *	1.58 (1.14–2.18) *	2.25 (1.51–3.36) *	0.61 (0.53–0.69) *	1.60 (1.53–1.67) *
Pre-frail	2.07 (1.09–3.92) *	1.48 (1.24–1.77) *	0.80 (0.35–1.80)	0.54 (0.15–1.87)	0.31 (0.17–0.45) *	1.28 (1.17–1.40) *
Frail	3.19 (1.64–6.17) *	2.27 (1.87–2.75) *	2.67 (1.27–5.63) *	2.87 (1.06–7.83) *	0.92 (0.74–1.09) *	2.15 (1.94–2.38) *
Severely Frail	10.84 (5.28–22.30) *	5.83 (4.54–7.48) *	3.47 (1.18–10.21) *	10.71 (3.57–32.17) *	2.84 (2.47–3.21) *	4.94 (4.19–5.83) *
MFI11						
Pre-frail	1.70 (0.55–5.32)	1.16 (0.86–1.57)	0.95 (0.42–2.13)	0.56 (0.16–1.91)	0.55 (0.11–0.99) *	1.25 (1.07–1.46) *
Frail	2.81 (0.89–8.88)	1.68 (1.21–2.32) *	2.51 (1.15–5.49) *	2.55 (0.92–7.12)	1.14 (0.86–1.95) *	2.24 (1.90–2.65) *
Severely Frail	7.68 (2.21–26.61) *	4.12 (2.80–6.23) *	3.16 (1.06–9.43) *	7.99 (2.51–25.40) *	4.01 (3.00–5.06) *	5.60 (4.29–7.32) *
mCCI	1.53 (0.35–1.74) *	1.35 (1.28–1.43) *	1.36 (1.10–1.68) *	1.60 (1.25–2.05) *	0.40 (0.34–0.46) *	1.30 (1.26–1.35) *
ASA	2.48 (1.06–1.10) *	2.54 (2.25–2.86) *	1.87 (1.13–3.09) *	1.88 (0.98–3.59)	1.02 (0.92–1.12) *	2.49 (2.32–2.66) *

MFI-5, MFI-11, Modified Frailty Index 5-point or 11-point. mCCI, Modified Charlson Co-morbidity Index. ASA, American Society of Anesthesiology Grade; * indicates the result is statistically significant (*p* value < 0.05).

**Table 7:** Area under Receiver Operating Characteristics curve analysis by model, for each outcome studied.

	Mortality	Major Complication (Pneumonia, DVT/PE, MI, Cardiac Arrest, Wound Infection/Dehiscence, Sepsis, CVA)	Unplanned Readmission	Unplanned Reoperation	Hospital Stay 30 Days or Greater after Index Case	Discharge to Non-Home Destination
Base model (approach, number of levels, gender)	0.68	0.68	0.74	0.77	0.69	0.72
Base model + Age	0.80	0.74	0.77	0.76	0.76	0.80
Base model + MFI5	0.77	0.74	0.78	0.82	0.73	0.78
Base model + MFI11	0.75	0.70	0.73	0.78	0.69	0.76
Base model + mCCI	0.81	0.75	0.79	0.80	0.78	0.80
Base model + ASA	0.76	0.75	0.76	0.78	0.79	0.79
Final multivariable model (age, MFI-5, approach, number of levels, gender)	0.83	0.76	0.79	0.84	0.78	0.81

MFI-5, MFI-11, Modified Frailty Index 5-point or 11-point; mCCI, Modified Charlson Co-morbidity Index; ASA, American Society of Anesthesiology Grade.
